# Microstructure and Mechanical Properties of Joints Depending on the Process Used

**DOI:** 10.3390/ma15155171

**Published:** 2022-07-26

**Authors:** Dawid Stanisz, Tomasz Machniewicz, Sławomir Parzych, Grzegorz Jeż, Leonid Dvorkin, Marek Hebda

**Affiliations:** 1 Faculty of Materials Engineering and Physics, Cracow University of Technology, Warszawska 24, 31-155 Kraków, Poland; d.stanisz@wisniowski.pl (D.S.); slawomir.parzych@pk.edu.pl (S.P.); 2 Wiśniowski Sp. z o.o. S.K.A., Wielogłowy 153, 33-311 Wielogłowy, Poland; g.jez@wisniowski.pl; 3 Faculty of Mechanical Engineering and Robotics, AGH University of Science and Technology, A. Mickiewicza Av. 30, 30-059 Kraków, Poland; machniew@agh.edu.pl; 4 Department of Building Materials Technology and Material Science, The National University of Water and Environmental Engineering, 11 Soborna St., 33028 Rivne, Ukraine; dvorkin.leonid@gmail.com

**Keywords:** arc welding MIG/MAG, laser welding, Cold Metal Transfer, joining thin-walled steel materials, robotic welding, butt welding

## Abstract

Today, numerous design solutions require joining thin-walled sheets or profiles as the traditional methods of welding with a consumable electrode in gas shielding, most often used in production processes, do not work well. The reason for this is that a large amount of heat is supplied to the joint, causing numerous welding deformations, defects, and incompatibilities. Moreover, the visual aspect of the connections made more and more often plays an equally crucial role. Therefore, it is important to look for solutions and compare different joining processes in order to achieve production criteria. The paper compares the properties of a 1.5 mm thick steel sheet joined by the manual and robotic MAG 135 and 138 welding process, manual and robotic laser welding, CMT welding with the use of solid or flux-cored wire, and butt welding. The macro- and microstructure, as well as the microhardness distribution of individual regions of the joints, were analyzed depending on the type of joining technology used. Furthermore, the mechanical properties of individual zones of joints were investigated with the use of a digital image correlation system. On the basis of the obtained test results, it was found that the joints made by the processes of manual laser welding and butt welding were characterized by a very regular weld shape, the smallest joint width, and greater grain refinement compared to other analyzed processes. Moreover, this method was characterized by the narrowest zone of hardness increase, only 3 mm, compared to, e.g., a joint made in the process of robotic welding CMT, for which this zone was more than twice as wide. Furthermore, the heat-affected zone for the joints made in this way, in relation to the welds produced by the MAG 135/138 method, was, respectively, 2 and 2.7 times smaller.

## 1. Introduction

Joining elements with a consumable electrode in arc welding Metal Inert Gas (MIG) or a consumable electrode in active gas Metal Active Gas (MAG) are leading methods in production processes. These methods enable high welding performance, easy inspection of the arc weld, the possibility of careful observation of the weld pool, the possibility of combining a wide range of materials, obtaining good mechanical properties of connections, and ease of mechanization and robotization of the welding process [1,2,3,4]. However, the traditional methods of MIG/MAG welding of steel materials with a thickness of less than 3 mm cause many technological problems. These are mainly related to the introduction of a large amount of heat to the welded joint, which can cause numerous welding deformations and distortions. Moreover, they can also lead to the formation of numerous welding spatters, thus lowering the aesthetics of manufactured products and increasing the laboriousness of finish machining of the detail after welding [5].

Today, more and more often in various industries, e.g., automotive, household appliances, or fencing systems, there is a necessity of making permanent joints of thin-walled steel elements, either without or with various coatings. Due to the above, in order to eliminate the previously described issues that arise when welding thin-walled elements, new variants of MIG/MAG welding have been developed, such as Cold Metal Transfer (CMT), Cold Arc, or Surface Tension Transfer (STT) [6,7,8]. In comparison to classic methods, the low-energy welding process MIG/MAG lends itself to welding defects reduction, a significant reduction in the amount of welding spatters, reduction in the amount of total energy implemented to the joint during welding, and reduction in harmful pollutants emission and thereby the improvement of the working conditions. As a result, they shorten the time and labor consumption of the production process and reduce cost-consuming technological procedures [9,10,11].

In addition, currently, the quality, precision, and aesthetics of welded joint designing are becoming more and more relevant for customers, which are essential, for example, during fence system production, door joinery, or the broadly understood furniture industry. Furthermore, the present trend observed is focused on solutions for the privacy of use, which can be obtained by reducing the translucency of fillings, for example, in fence systems. However, the use of classic components would result in a significant increase in the weight of the product, which, in turn, would necessitate the use of special fittings and more powerful drives. Therefore, it is necessary to reduce the cross-sections of the profiles used and the thickness of the sheets in order to minimize the weight of the product, as well as the operating and assembly costs. However, the aesthetics of individual products depends not only on the welding method used, but also on the process parameters, experience and knowledge of employees, the use of additional technological procedures, and even the sequence of connections.

Another method of joining materials that is more often used in the industry is the laser welding process, with or without additional material. However, it should be noticed that due to the specific heat source, which is the laser beam, the welding process itself and the metallurgical processes taking place during welding are significantly different from the processes taking place in arc welding methods. As a consequence, the properties of welded joints will be different from those obtained when using conventional technologies [12,13,14]. One of the latest solutions in the field of laser welding is the dynamically developing devices for manual welding in recent years, which use a beam of laser radiation generated by sources with a power of 1kW to 2kW [5,15,16]. However, at the moment there is a lack of precise information regarding the safety of employees in the workplace equipped with this type of equipment.

The processes of joining thin-walled steel materials can also be carried out using various resistance welding technologies [17,18]. This method is characterized by low operating costs and no need to use additional materials. As a result, welding does not cause significant changes in the chemical composition and metallurgical properties, while maintaining the mechanical properties. Additional advantages of the resistance welding processes are the speed of making connections, simplicity of the course, and the possibility of remote control. However, it should be remembered that when welding thin-walled elements, there are great difficulties with ensuring dimensional tolerances of the structures made, which are related to the need to select an appropriate technological allowance and the upset speed of the joined elements. The appropriate selection of process parameters, such as current intensity and welding pressure force, enables the minimization of welding deformations and deformations [19,20]. In addition, welding allows you to connect two elements with a large variation in the thickness of the materials to be joined, which is very difficult or sometimes even impossible to achieve based on the MIG/MAG arc welding processes.

The literature contains only residual information presenting a comparison of the properties of inseparable connections of thin-walled steel elements made with the use of various bonding methods. Therefore, the article presents the results of research on the strength properties and metallographic structures of joints made by the following methods: MAG (135 and 138), CMT (using solid or flux-cored wire), laser welding, and butt welding. In addition, the results of manual and robotic welding (for the MAG and laser welding methods) were compared. In addition, the article also presents the possibilities of using the digital image correlation (DIC) system to analyze the strength properties of joints and joint zones depending on the method used.

## 2. Materials and Methods

The welding process was analyzed for cold-rolled low-carbon steel 1.0330 (DC01), 1.5 mm thick and chemical composition according to EN10130/EN10139 C ≤ 0.12, Mn ≤ 0.60, P ≤ 0.045 and S ≤ 0.045. The strength parameters of the base material were determined in the static tensile test. These parameters reached the following values: Rm = 317 MPa and R_0.2_ = 228 MPa. Table 1 presents a list of the analyzed welding methods along with the process parameters used. The samples were prepared and made in accordance with the guidelines of PN-EN ISO 15614-1 and PN-EN ISO 15614-11.

Samples welded manually using the MAG 135 and MAG 138 methods were made at a stationary welding station using the inverter MIG/MAG Powertec i320c Advanced welding device (Lincoln Electric Bester, Bielawa, Poland). The MAG 135 process was carried out with the use of an electrode in the form of a solid wire EN ISO 14341-A: G 38 3 C1 3Si1 with a diameter of 1.0 mm. In the MAG 138 process, an electrode in the form of an EN ISO 17632-A-T 46 4 M M 2 H5 metallic core with a diameter of 1.2 mm was used. The shielding gas for both processes was a mixture of 92% Ar 8% CO_2_ according to PN-EN ISO 14175-M20-ArC-8, with a constant shielding gas flow rate 12 l/min.

The samples made using the MAG 135/138 and CMT robotic processes were prepared on a welding station equipped with a Kawasaki RA006L (Kawasaki Robotics, Lakeview Drive, Wixom, MI, USA) an industrial robot and Fronius TPS 400i MIG/MAG welding sources (Fronius International GmbH, Wels, Austria). The MAG 135 robotic process was carried out using an electrode in the form of a solid wire EN ISO 14341-A: G 38 3 C1 3Si1 with a diameter of 1.2 mm, and in the MAG 138 process, an electrode in the form of a wrapped wire with a metallic core was used EN ISO 17632-A-T 46 4 M M 2 H5 with a diameter of 1.2 mm. For the CMT process, electrodes in the form of a solid wire EN ISO 14341-A: G 38 3 C1 3Si1 with a diameter of 1.2 mm and a cored wire with a metallic core EN ISO 17632-A: T 42 4 ZMn M M21 1 H5 were used. For all robotic processes, the shielding gas was 92% Ar 8% CO_2_ according to PN-EN ISO 14175-M20-ArC-8, with a constant shielding gas flow rate 14 l/min.

Laser welding was performed in the research laboratory of IPG Photonics in Gliwice. The laser welding process without additional material was carried out on a robotic station equipped with an ABB industrial robot and a laser source with a power of 6 kW. The process was carried out without a gas shield. On the other hand, samples with welding consumables were made in the process of manual laser welding using the LightWeld XC device by IPG Photonics (Oxford, MA, USA) The manual laser welding process was carried out with the use of an electrode in the form of a solid wire EN ISO 14341-A: G 38 3 C1 3Si1 with a diameter of 1.0 mm, in a welding gas shield with nitrogen N50, with a constant flow rate of the shielding gas 25 l/min.

The process of resistance butt welding was carried out using the ZDZ7 butt-short-circuit welding machine (ASPA, Wrocław, Poland).

**Figure 1:**
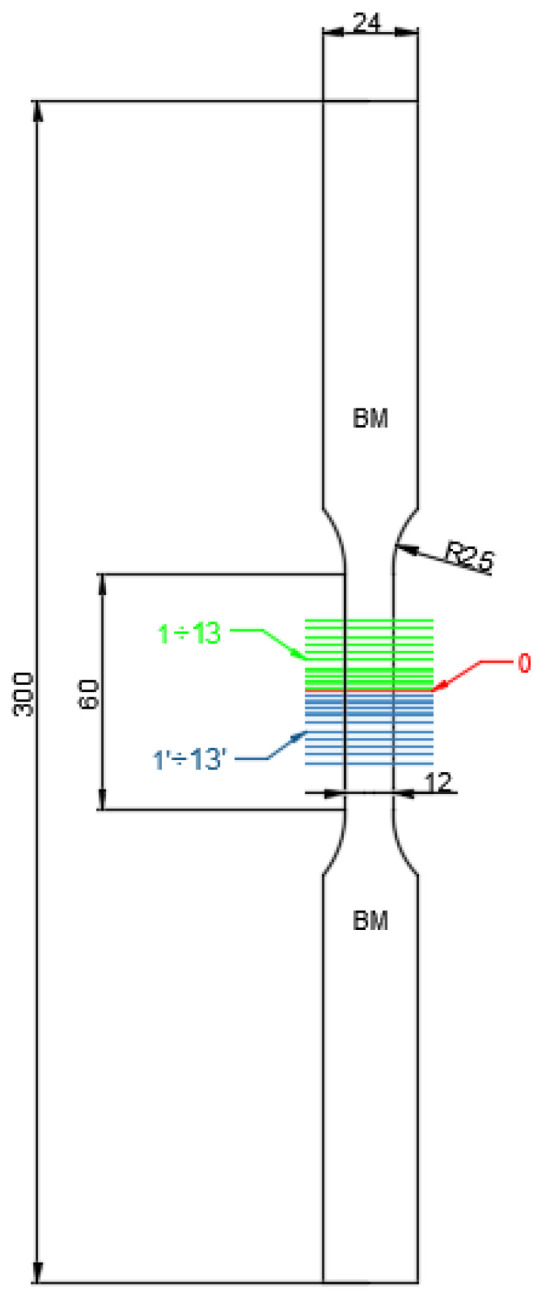
The geometry of samples for the static tensile test of thin-walled steel elements bonded by various methods. Designation of areas used for analysis using the digital image correlation system (DIC): 0—a center of the welded joint, BM—based material, 1÷13—an area of the analyzed sample from the weld axis upwards, and 1'÷13'—an area of the analyzed sample from the axis weld down (Unit: mm).

**Figure 2:**
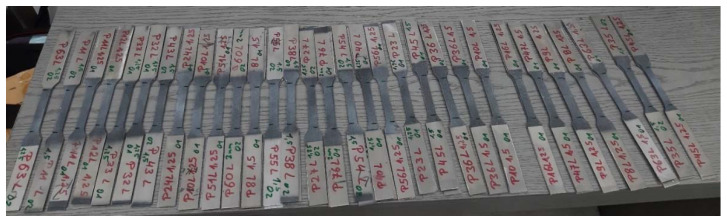
A photo of specimens prepared for the static tensile test with the use of a digital image correlation system (DIC).

Static tensile tests of the manufactured joints were carried out on samples with the geometry shown in Figure 1 and Figure 2, using an MTS 810 machine (MTS Systems Corporation, Eden Prairie, MN, USA) equipped with a digital image correlation (DIC) system. Tensile tests were carried out with a displacement rate of 2 mm/min, until destruction.

**Figure 3:**
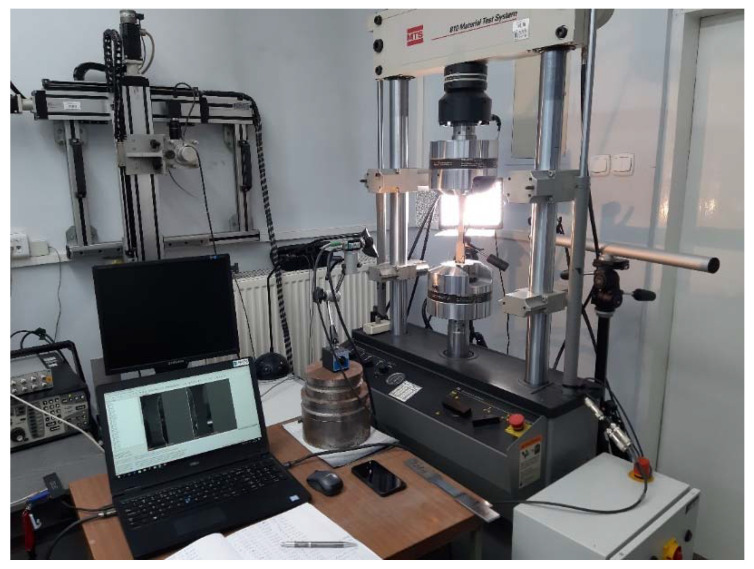
Stand for static tensile tests with simultaneous analysis of sample deformation based on the digital image correlation system (DIC), simultaneously recording the image of both flat surfaces of the tested sample.

The deformation analysis of the area of welded joints was performed using the Dantec Dynamics Istra 4D (version 4.7.0.495) [21] digital image correlation system (DIC). A system of two cameras with a resolution of 5 Mpx placed opposite to each other was used, simultaneously recording the image of both flat surfaces of the tested sample (Figure 3). Prior to testing, stochastic speckles of an average physical size of 0.3 mm were produced on the surface of the samples by spraying black and white paint. In combination with the resolution of the cameras and the zoom of the lens, this corresponded to the recommended spot sizes of 3–5 pixels [22,23]. The speed of the image acquisition was 2 Hz, which, in combination with the speed of the test, corresponded to one frame for the extension of the sample by 1.7 µm. The analysis of the deformation fields was carried out with the use of the Istra 4D [21] program, with the face size of 17 pxs and the mesh spacing of 13 pxs [21,22].

**Figure 4:**
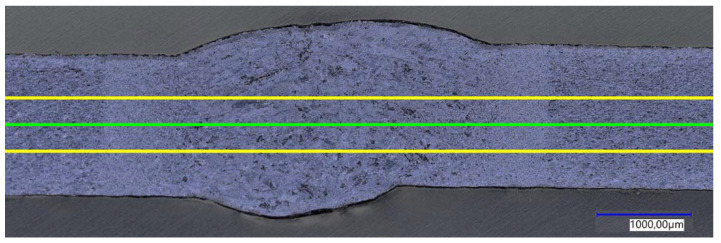
Representative diagram of the microhardness measurement locations: in the sample axis—green line, at a distance of 0.25 mm from the sample axis—yellow lines.

The cross-sections of the welded joints were subjected to the microscopic analysis (Keyence VHX-7000, Osaka, Japan) and microhardness measurements using the Vickers HV 0.5 kg (49.1 N) method (TUKON 2500 by Willson Instruments, Flums, Switzerland). The microhardness was analyzed along three parallel lines (Figure 4).

Measurements were started in the unaffected base material, covered the weld zone, and ended again in the unaffected base material. The first measurement was made in the axis of the joined sheets, and the next ones were symmetrically distant from the axis by 0.25 mm. Measurements were carried out in 0.5 mm intervals.

## 3. Results and discussion

**Figure 5:**
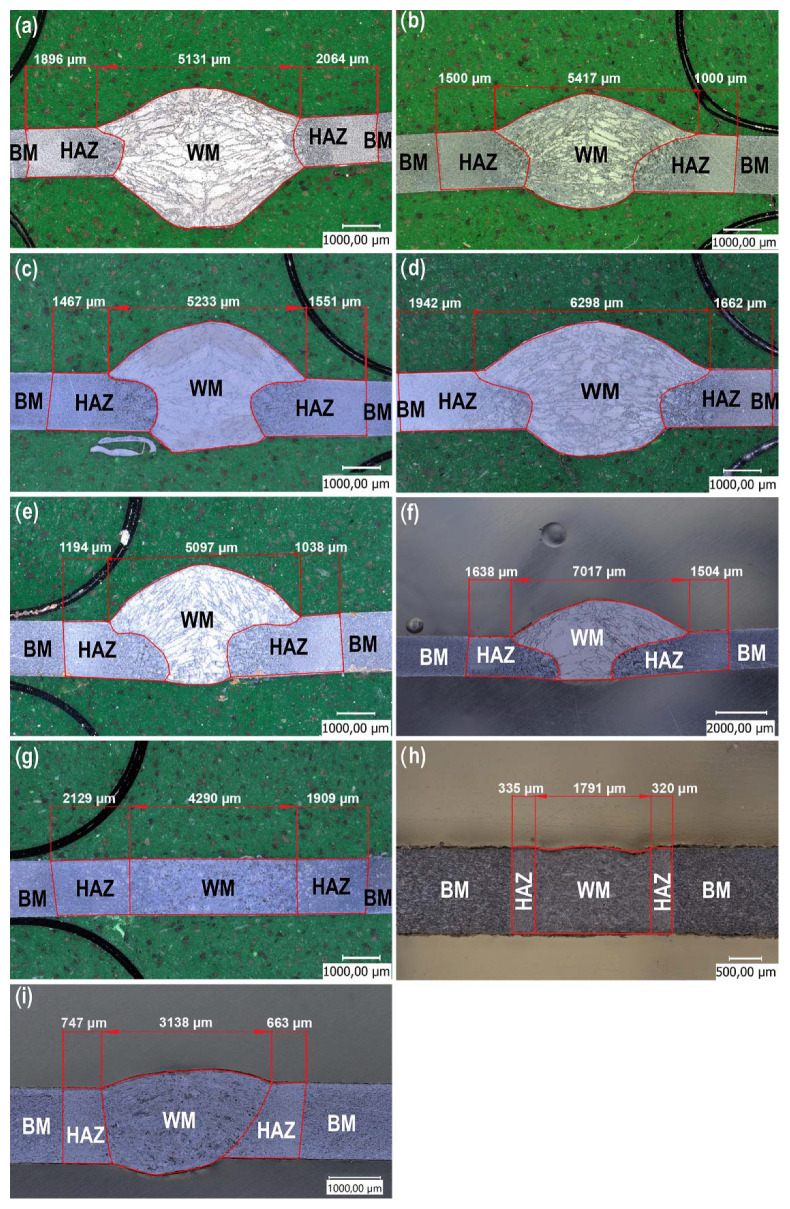
Representative macrostructures of cross-sections of joints depending on the bonding process used: (**a**) manual MAG135, (**b**) manual MAG138, (**c**) robotic MAG135, (**d**) robotic MAG138, (**e**) CMT solid wire, (**f**) CMT flux cored wire, (**g**) butt welding, (**h**) robotic laser, (**i**) manual laser with solid wire.

Figure 5 shows representative microstructures of the tested joints. Regardless of the method of joining thin-walled steel elements, characteristic weld zones were observed in each case: weld metal (WM), heat effect zone (HAZ), and base material (BM) [24,25]. The made connections were free from welding imperfections and structural defects, e.g., material discontinuities. It was found that the shape of the welds obtained after welding with MIG/MAG methods has a characteristic appearance resembling the so-called “mushrooms”. Moreover, the joints made with metallic core wires were characterized by a more regular weld profile, which, consequently, resulted in much smaller deformations in the joined sheets.

The smallest width of the weld and heat-affected zone (HAZ) was obtained in the process of robotic laser welding without the addition of material. Compared to traditional MAG 135/138 welding methods, they were more than three times smaller. Moreover, the processes of manual laser welding and butt welding were characterized by smaller areas of joints compared to the MAG 135/138 method, by 2 and 2.7 times, respectively. This property is of great importance in the context of minimizing the longitudinal deformation of the welded elements, which results from the occurrence of longitudinal shrinkage. It is related to the reduction in the volume of the weld in relation to other methods and the reduction in the ratio of the surface area of the joined elements to the cross-sectional area of the welds obtained. The direction of contraction of the joint follows the direction of its axis. As a result of thermo-mechanical changes in the weld and in the heat-affected zone, the contraction force is distributed over the entire cross-section of the element. The plane of this section is perpendicular to the axis of the welds. The greater the ratio of the cross-sectional area of the element to the cross-sectional area of the welds in a given plane, the lower the longitudinal shrinkage. The force of contraction is distributed over a larger area and thus the longitudinal elastic deformation of the element will be smaller [26,27].

It was also observed that the application of these processes allows us to obtain a very regular profile of the joint, which in turn translates into negligible deformation of the joined elements. It is an important parameter in the case of bonding thin-walled elements in order to minimize welding deformations of the construction [26,27].

The macroscopic analysis of the structures showed that the joints made by classical welding and laser welding were characterized by a weld face width of 2.5–3.6 mm, which was about 50% of the width of joints made using semi-automatic methods. Moreover, it was observed that for the CMT process (Figure 5e,f), the fusion width was much smaller compared to the other joints. Furthermore, the joint made by manual laser welding with the use of filler material was of similar width to the joint made by butt welding (Figure 5g) or robotic laser welding (Figure 5h).

**Figure 6:**
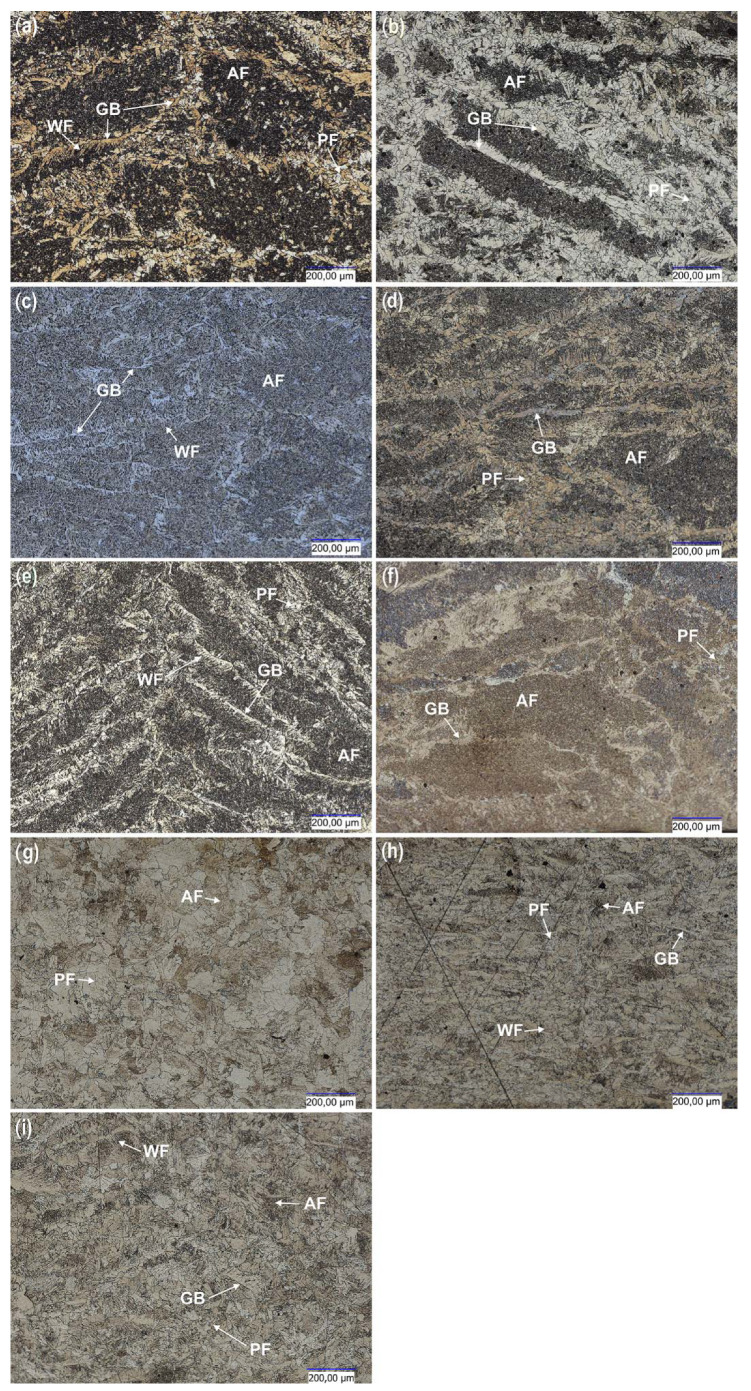
Representative microstructures of cross-sections of joints depending on the bonding process used: (**a**) manual MAG135, (**b**) manual MAG138, (**c**) robotic MAG135, (**d**) robotic MAG138, (**e**) CMT solid wire, (**f**) CMT flux cored wire, (**g**) butt welding, (**h**) robotic laser, (**i**) manual laser with solid wire.

Figure 6 shows the representative microstructure of the welds depending on the bonding processes. Depending on the process used, acicular ferrite (AF), polygonal ferrite (PF), boundary ferrite (GBF) and Widmanstatten ferrite (WF) [26,28] can be observed in the microstructures.

The largest size of the primary grain of austenite and GBF, PF, WF occurs in the sample welded by the manual method 135 and 138 (Figure 6a,b). In the case of welds made with the use of flux-cored wires and a robotic station, a high content of AF, GBF was noticed (Figure 6c,d). The CMT samples (Figure 6e,f) had a smaller size of the primary austenite grain and amount of PF compared to manual MAG 135 and MAG 138 (Figure 6a,b). Moreover, more AF ferrite was found for the CMT method (Figure 6e,f). In the case of using the welding and laser welding processes (with and without filer material), no GBF was observed but only AF and PF (Figure 6h–j). Additionally, the smallest grain was visible in these samples.

**Figure 7:**
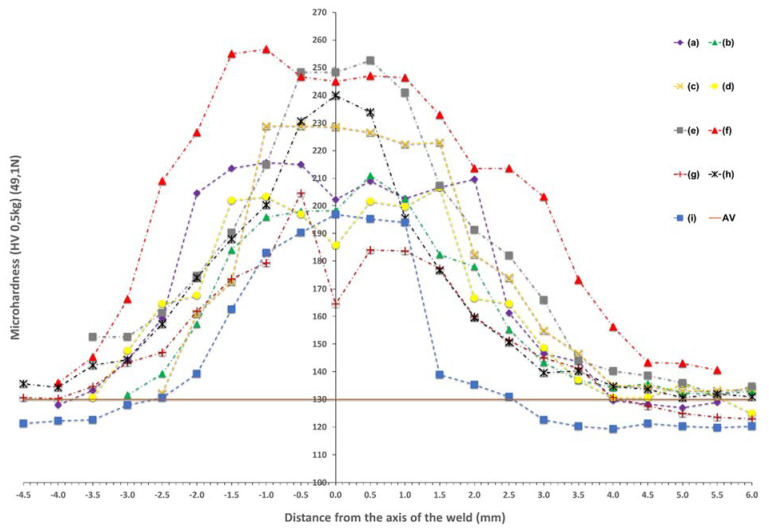
Distribution of the microhardness of the joints depending on the bonding process used: (a) manual MAG135, (b) manual MAG138, (c) robotic MAG135, (d) robotic MAG138, (e) CMT solid wire, (f) CMT flux-cored wire, (g) butt welding, (h) robotic laser, (i) manual laser with solid wire, AV - the average value of the microhardness of the base material.

Analyzing the microstructure of the welds in Figure 6e,f it can be concluded that both welds contain Widmanstatten ferrite (FW, nucleating at the boundaries of the former austenite grain). This means that in both cases the thermodynamic conditions for the formation of this microstructure were met. Estimating the amount of this ferrite based on the observation of the microstructure, it can be assumed that in the weld in Figure 6e (made with a solid wire) there is slightly more FW ferrite than in the weld made with a flux-cored wire (Figure 6f). When analyzing the documentation of the manufacturers of binders (used in the experiment) regarding the chemical composition of welds after MAG processes, it can be noticed that the weld made with flux-cored wire contains more manganese by as much as 1/3 (1.52% by weight) compared to solid wire (0.94% wt). Since manganese promotes the formation of FW ferrite, a slightly higher amount of this ferrite in the weld with a lower manganese content may suggest that other factors had a greater influence on the formation of this phase. In this case, the cooling speed can be considered, which was probably lower for a joint made with a flux-cored wire (Figure 6f). This may be indicated by the width of the heat affected zone and the zones of increased hardness, which are much larger for this joint than for a joint made with a solid wire (Figure 6e), as shown in Figure 7. The differences in the kinetics of FW formation are also indicated by its morphology. The ferrite in the weld from Figure 6e has a greater share of the needle zone in relation to the FW nucleation area. On the other hand, the needles of the FW shown in Figure 6f are less exposed, which suggests a greater tendency for longer growth with a stable transformation front and destabilization only at the final stage, when the needles were formed. Referring also to the remaining part of the microstructure, i.e., the space inside the former austenite grain, it can be seen that the microstructure in the weld obtained with the use of a flux-core wire (Figure 6f) is finer, where acicular ferrite is probably present. It should be remembered that with a carbon content of approx. 0.1% by weight (according to catalog data), carbon also has its share in the microstructure, in which it will mainly be deposited together with coniferous ferrite inside the former austenite grain. However, based on studies using only a light microscope, its role in morphology has not yet been determined. It can only be assumed that due to the probable presence of coniferous ferrite in these areas, the microstructure will be bainitic [26,27,28,29,30].

The above-described changes in the microstructure are reflected in the results of microhardness measurements on the cross-section of the joints presented in Figure 7. It was observed that regardless of the method used, all the registered microhardness profiles within the welded joints made have a characteristic shape of a symmetrical normal distribution, typical for steel welded joints. The highest values of microhardness were always recorded in the central zone of FZ of the weld [24,25]. This effect was independent of the side from which the analyses were carried out (face, ridge) [31,32,33].

In general, it was observed that the shape of the microhardness distribution on the cross-section of the joints was similar, regardless of the bonding method used. The measured differences in microhardness were a consequence of the joining process used: manual, CMT, laser, and solid and flux-cored wire. Moreover, for the MAG 135 and 138 processes, in which the additional material was used, the difference in the recorded microhardness values was caused by the different content of Si and Mn, which influenced the solution hardening resulting in the increase in microhardness [28,29,30].

Very similar widths of zones with increased hardness were also observed for joints made in the arc welding processes (MIG/MAG and CMT), with simultaneous different hardness values between joints made with additional material in the form of solid wire, and joints made with the addition of flux-cored wire. The hardness values of the welds for individual processes ranging from 10 to 20 (HV). The exceptions were samples made in the CMT process, for which the maximum hardness values are the same, while for solid wire they reach values similar to the hardness of the base material much faster. This phenomenon is due to the fact that the synergic line for the CMT process has been developed by Fronius dedicated to solid wires. The CMT method in combination with metal cored wires is characterized by a lack of stability in the process parameters and the inability to achieve a welding speed similar to solid wires. The conducted tests show that it would be necessary to develop a special synergic line dedicated to flux-cored wires, without which, for the welded joints made, there is a wide zone of increased microhardness and a diversified microstructure.

Among all the analyzed processes, the widest zone of hardness increase was noted for the joint made in the process of resistance butt welding, for which the maximum values were comparable to the hardness values obtained in the process of welding with flux-cored wires. This phenomenon may result from the course of the resistance butt welding process, in which the joined elements are heated at the same time over their entire contact surface. In addition, the length of the electrode attachment zone is responsible for the width of the material heating zone, which depends on the type of materials to be joined. The welding area is heated to plasticize. When the metal in the welding zone reaches the required temperature, it undergoes characteristic swelling under the influence of the pressure force, and the contacting surfaces join. The welding current is automatically switched off even before the upsetting process is completed while maintaining the pressure force. The applied pressure force introduces significant stresses in the welded joint, limiting the grain growth. This process course in combination with the uniform and gradual cooling of the joint under ambient conditions may have an impact on the width of the heat-affected zone and the zone of increased hardness. The stability of the process may also be demonstrated by the correctly columnar arrangement of grains and the fine-grained structure obtained, consisting mainly of acicular ferrite and polygonal ferrite. 

On the other hand, the narrowest zones of hardness increase were obtained for joints made in the laser welding processes, which is associated with the use of the high energy density of the laser beam. The comparison of the robotic and manual laser welding processes made it possible to show a large difference between the obtained results of the maximum weld hardness, amounting to about 50 (HV) more in the robotic process. Moreover, it was observed that for the samples made in the process of manual laser welding with the additional material, the smallest changes in hardness in the area of the entire weld were obtained. The reduced hardness of the heat-affected zone, in relation to the other considered variants, may be associated with more far-reaching processes of microstructure renewal with regard to the reduction in the dislocation density. In iron-based alloys, especially in low-carbon steels, this process is generally accomplished by achieving the thermodynamic conditions of transformation into austenite and another transformation into a ferrite-based structure [28,29]. It should be noted, however, that the discussed variant of welding (manual laser) involves the use of high energy density in a less stable way (manual process control). Large temperature gradients accompanying heating by the laser beam, combined with the variability of the amount of energy at a given point of the joint, can cause a very rapid temperature increase and subsequent rapid cooling [34]. Bearing in mind that the acceleration of the rate of temperature changes also causes a change in the temperature at which the phase changes take place, it is also possible to consider the variant that there is no typical kinetics of changes in the HAZ, and it is possible not to obtain a regular austenite grain at the HAZ showing low hardness [28,29,30,34]. In addition, the analyzed material is cold-rolled steel, and therefore contains a high density of dislocation, which allows us to analyze the possibility of healing or even recrystallization processes in a more classic approach. Of course, it should be noted that high-strain recrystallization leads to grain refinement, and, thus, material strengthening, and requires thermodynamic conditions for nucleation and new grain growth, which may be difficult to achieve with rapidly changing processes [26,27,28,29,30]. Verification of the indicated hypotheses is possible through more detailed microstructure studies, especially with the use of transmission electron microscopy (TEM), which is planned at the next stages of the research. The analysis of the microstructure at such a level will provide answers regarding the mechanisms of material strengthening or the lack of them.

**Figure 8:**
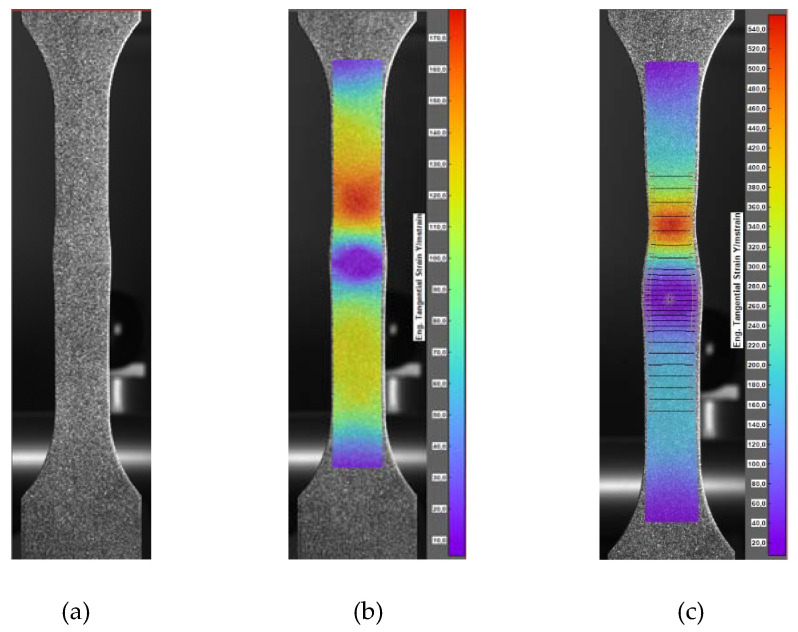
Phases of sample image analysis with the use of Istra 4D software: (**a**) image of the sample with specks, (**b**) image of the sample with the deformation field, (**c**) image of the sample with the deformation field, including lines defining the cross-section planes in which the tension curves were determined.

**Figure 9:**
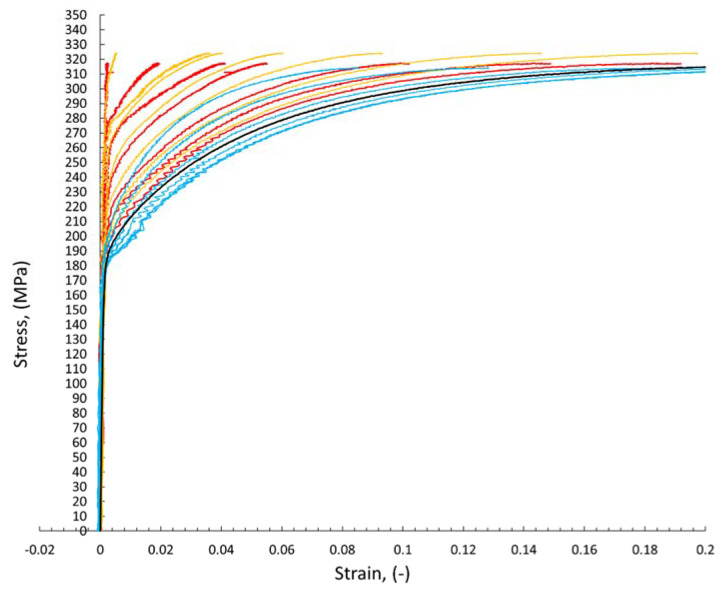
The curves of the static tensile test determined on the basis of the DIC method for welded joints made in robotic welding processes: red—MAG 135; orange—MAG 138; blue—laser welding without additional material; black—base material.

**Figure 10:**
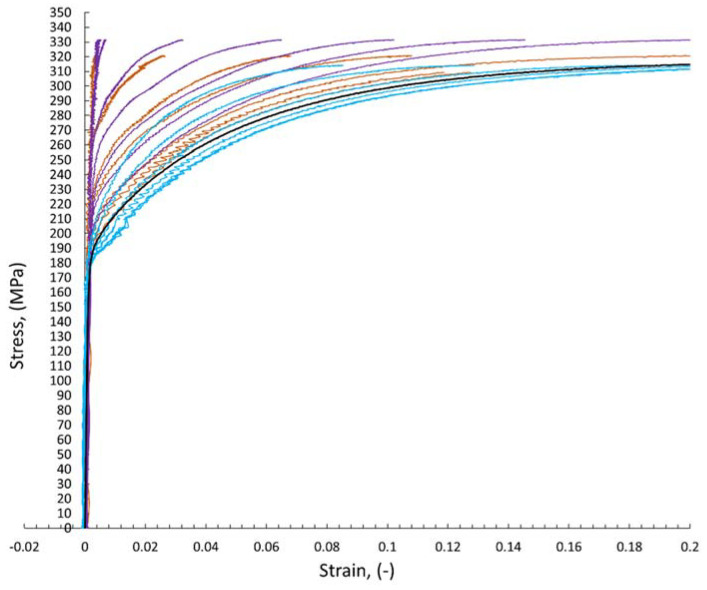
The curves of the static tensile test determined on the basis of the DIC method for welded joints made in robotic welding processes: brown—CMT solid wire; violet—CMT flux-cored wire; blue—laser welding without additional material; black—base material.

**Figure 11:**
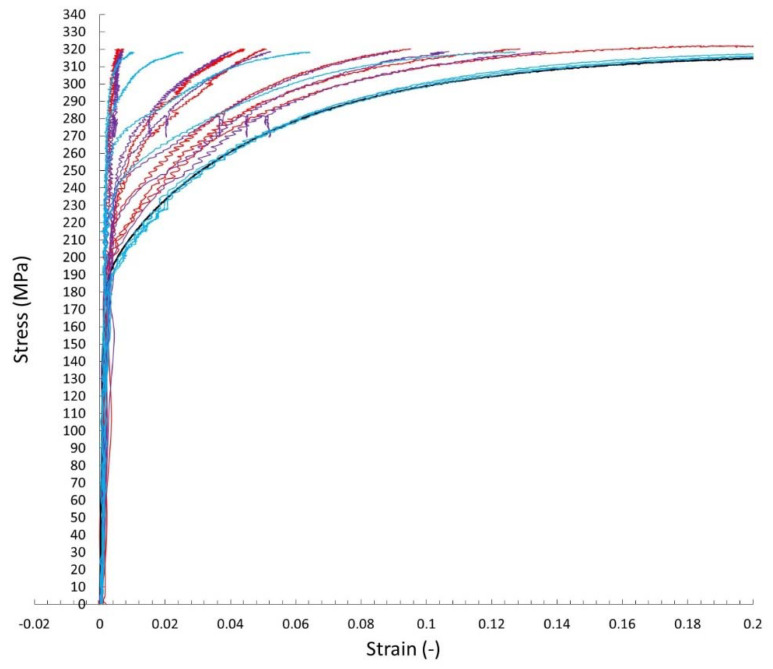
The curves of the static tensile test determined on the basis of the DIC method for welded joints made in manual welding processes: red—MAG 135; purple—MAG 138; blue—manual laser welding with additional material; black—base material.

The digital image correlation system (DIC) was used to assess the differences in mechanical properties occurring during static tensile tests for individual areas of each of the analyzed joints. This enabled the determination of tensile curves for individual sections of welded joints in planes transverse to the axis of the samples under tension. Figure 8 shows an exemplary representative image of the sample at individual stages of its analysis using the Istra 4D software. Moreover, Figure 9, Figure 10 and Figure 11 show the curves determined by the DIC system for robotic and manual samples, respectively. The values of axial deformation for a given location within the scope of the weld were determined along the same lines indicated on both surfaces of the samples (i.e., on both sides of it) in the same cross-sections distant by 1 mm. In order to take into account the possible secondary bending effect, the deformations in the section under consideration were calculated as the average of the values determined on both sides of the sample [31].

It is well known that the yielding and strain hardening behavior is determined by hardness distribution across the weld joints. Figure 9, Figure 10 and Figure 11 demonstrate the consistent trends of the increase in plastic strains in the cross-sections of the welded specimens with increasing the distance from the weld center, which indicates a decrease in the yield stress for subsequent zones. This type of trend is characteristic of fusion joints [35]. The changes in the yield point were in each of the considered welding techniques symmetrical around the weld center line, which is qualitatively different compared, for example, to friction stir welds [31,36]. All the welded specimens ruptured in the cross sections away from the fusion zone, as shown in Figure 8c For the material from weld center sections, the 0.2% proof stress (R_0.2_) was not reached before specimen fracture, which indicates that it was higher than the ultimate tensile strength of the base material. The effect of the yield stress decreasing, visible in Figure 9, Figure 10 and Figure 11, qualitatively corresponds very well with the microhardness changes presented in Figure 7.

**Figure 12:**
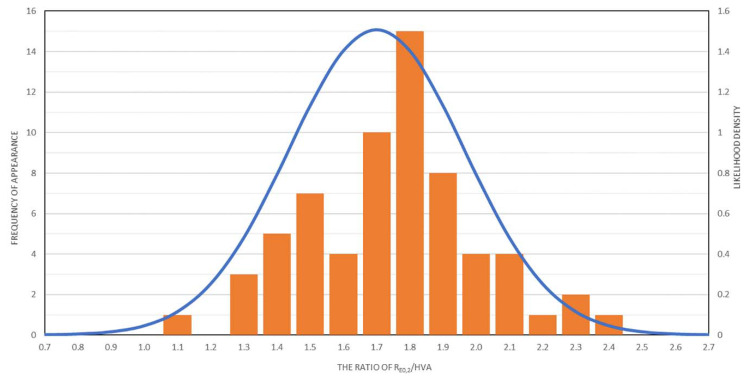
The relationship between the microhardness values and the conventional yield strength Re_0.2_ depending on the joining method used.

On the basis of the obtained results, the relationship between the microhardness values and the conventional yield point Re_0.2_ was determined, depending on the method of producing welded joints. The R_0.2_/HVA ratio for the base material was 1.75. The obtained results are presented in Figure 12. On their basis, it is possible to indirectly determine the local values of the mechanical parameters of the joint.

Based on the data presented in Figure 6, it can be concluded that the microhardness of the material (HVA) decreased with increasing distance from the weld axis. This phenomenon was accompanied by an increase in the strength parameters represented by the yield point R_0.2_ (Figure 9, Figure 10 and Figure 11). However, due to the different bonding techniques contemplated herein, including the different types of welding wire used, the R_0.2_/HVA ratios determined for the different welds and different connection zones have varied over a fairly wide range. As shown in Figure 12, which includes the results of all measurements in total, the R_0.2_/HVA ratio ranged from 1.1 to 2.4, which could be described by a Gaussian curve, with a mean value 1.7 and a standard deviation 0.26. The relatively large range of changes in this ratio does not allow us to conclude the mechanical characteristics of the material only on the basis of the microhardness. Determining such a relationship would have to be limited to only one joining technique [26,31].

## 4. Conclusions

On the basis of the obtained results of various steel joining methods, it was found that the narrowest zone of hardness growth, approximately 3 mm, was obtained for joints made by laser welding. This process was characterized by high dynamics and high energy density concentrated in a small area of connected materials. Therefore, there was also no growth of microstructure grains, which is a standard phenomenon observed for other joining processes, introducing large amounts of thermal energy to the joints. On the other hand, the widest zone of hardness increase, about 7 mm, was recorded for a joint made in the CMT robotic welding process. Moreover, this method, regardless of the type of wire used, solid or flux-cored, gave the highest microhardness values. It was found that the manual laser welding and butt-welding processes were characterized by smaller areas of joints compared to the MAG 135/138 method. Furthermore, the heat-affected zone for the joints made in this way, in relation to the welds produced by the MAG 135/138 method, was, respectively, 2 and 2.7 times smaller. Joints made by butt welding and laser welding processes were characterized by a weld face in the range of 2.5–3.6 mm, which was about 50% of the width of welds made using robotic methods. In addition, the butt welding processes and, in particular, manual laser welding, were characterized by a uniform distribution of stresses in the welded joint, which, in turn, resulted in minimizing the deformation of the joined elements. This parameter is particularly important in the case of joining thin-walled structures and indicates great potential for using these processes as an alternative to conventional welding processes used in the industry.

## Figures and Tables

**Table 1:** Methods of welding thin-walled steel elements with the applied process parameters.

**The methods of welding**	**Process parameters**					
	**Welding current (A)**	**Welding voltage (V)**	**Wire feed speed (m/min)**	**Time of welding (sec)**	**Speed of welding (cm/min)**	**Heat input** **(kJ/cm)**
MIG/MAG						
MAG 135 manual	120	18.1	7.1	47.0	44.7	2.33
MAG 138 manual	70	17.0	7.1	35.0	60.0	0.95
MAG 135 robotic	167	17.0	5.1	33.2	63.3	2.15
MAG 138 robotic	144	15.8	3.8	33.3	63.1	1.73
CMT solid wire	195	16.1	6.2	15.0	140.0	1.07
CMT flux-cored wire	160	13.3	4.0	34.0	61.8	1.65
**Laser welding**						
	**Laser beam power (W)**	**Oscillation (mm)**	**Wire feed speed (m/min)**	**Time of welding (sec)**	**Speed of welding (cm/min)**	**Heat input** **(kJ/cm)**
Laser robotic without additional material	1800	0.6	-	12.0	150	0.43
Laser manual with additional material	1050	1.5	0.65	27.7	65	0.58
	**Welding**					
	**Welding current** **(kA)**	**-**	**-**	**-**	**Butt welding displace (mm)**	**Heat input** **(kJ)**
Resistance upset butt welding	5.8	-	-	-	4	6.728

## Data Availability

Not applicable.
